# *Brassica juncea BRC1*-1 induced by SD negatively regulates flowering by directly interacting with BjuFT and *BjuFUL* promoter

**DOI:** 10.3389/fpls.2022.986811

**Published:** 2022-09-30

**Authors:** Junjie Feng, Qinlin Deng, Huanhuan Lu, Dayong Wei, Zhimin Wang, Qinglin Tang

**Affiliations:** ^1^ College of Horticulture and Landscape Architecture, Southwest University, Chongqing, China; ^2^ Chongqing Key Laboratory of Olericulture, Chongqing, China

**Keywords:** *BjuBRC1*, BjuFT, *BjuFUL*, flowering, *Brassica juncea*

## Abstract

Flowering is crucial for sexual reproductive success in angiosperms. The core regulatory factors, such as *FT*, *FUL*, and *SOC1*, are responsible for promoting flowering. *BRANCHED 1* (*BRC1*) is a TCP transcription factor gene that plays an important role in the regulation of branching and flowering in diverse plant species. However, the functions of *BjuBRC1* in *Brassica juncea* are largely unknown. In this study, four homologs of *BjuBRC1* were identified and the mechanism by which *BjuBRC1* may function in the regulation of flowering time was investigated. Amino acid sequence analysis showed that BjuBRC1 contained a conserved TCP domain with two nuclear localization signals. A subcellular localization assay verified the nuclear localization of BjuBRC1. Expression analysis revealed that *BjuBRC1*-1 was induced by short days and was expressed abundantly in the leaf, flower, and floral bud but not in the root and stem in *B. juncea*. Overexpression of *BjuBRC1*-1 in the *Arabidopsis brc1* mutant showed that *BjuBRC1*-1 delayed flowering time. Bimolecular fluorescent complementary and luciferase complementation assays showed that four BjuBRC1 proteins could interact with BjuFT *in vivo*. Notably, BjuBRC1 proteins formed heterodimers *in vivo* that may impact on their function of negatively regulating flowering time. Yeast one-hybrid, dual-luciferase reporter, and luciferase activity assays showed that BjuBRC1-1 could directly bind to the promoter of *BjuFUL*, but not *BjuFT* or *BjuSOC1*, to repress its expression. These results were supported by the reduced expression of *AtFUL* in transgenic *Arabidopsis* overexpressing *BjuBRC1*-1. Taken together, the results indicate that *BjuBRC1* genes likely have a conserved function in the negative regulation of flowering in *B. juncea*.

## Introduction

Flowering represents a crucial developmental transition from vegetative to reproductive growth, which is crucial for sexual reproductive success in angiosperms. The molecular mechanism and genetic basis of flowering have been characterized in detail in *Arabidopsis thaliana* (Andrés and Coupland, 2012; Dally et al., 2014; Blümel et al., 2015). Flowering is induced by complex exogenous and endogenous cues, such as photoperiod and hereditary factors. Six major pathways, comprising the autonomous, photoperiod, vernalization, aging, thermosensory, and gibberellin (GA) signaling pathways (Simpson and Dean, 2002; Blázquez et al., 2003; Halliday et al., 2003; Amasino, 2004; Han et al., 2008; Kumar et al., 2012; Fornara et al., 2010), regulate flowering by influencing the floral integrator genes *FLOWERING LOCUS T* (*FT*), *SUPPRESSOR OF OVEREXPRESSION OF CONSTANS 1* (*SOC1*), and *LEAFY* (*LFY*).

The FT protein is a phosphatidylethanolamine-binding protein (PEBP). The PEBP family consists of three phylogenetically distinct groups: FT-like proteins, TERMINAL FLOWER1-like (TFL1), and MOTHER OF FT AND TFL1-like (MFT) proteins (Pin and Nilsson, 2012; Wickland and Hanzawa, 2015). FT is transported from leaves to the shoot apex to induce flowering by forming a protein complex with the bZIP transcription factor FLOWERING LOCUS D (Wigge et al., 2005; Corbesier et al., 2007; Tamaki et al., 2007; Wickland and Hanzawa, 2015; Zhang et al., 2016) and is regulated under long days (LD) by transcription factors such as *CONSTANS* (*CO*) and *TEMPRANILLO* (*TEM*) (Castillejo and Pelaz, 2008). Furthermore, TFL1 and FT compete with FD and thus affect flowering (Zhu et al., 2020). Studies of tomato and rice indicate the involvement of 14-3-3 proteins in the protein interactions of FT orthologs with FD orthologs (Pnueli et al., 2001; Taoka et al., 2011). The MADS-box gene *FRUITFULL* (*FUL*) plays an essential role in regulating flowering time, differentiation of floral meristems, and carpel and fruit development in *Arabidopsis* (Ferrándiz et al., 2000; Melzer et al., 2008; Wang et al., 2009). In addition, *FUL* coordinates with the aging pathway and gibberellin signaling pathway to accelerate flowering (Yamaguchi et al., 2009; Li et al., 2016).

The TCP transcription factor family was first described in 1999. The conserved domain consists of a 59-amino acid basic helix–loop–helix (bHLH) motif that allows DNA binding and protein–protein interactions. The first characterized members were TEOSINTE BRANCHED 1 (TB1), CYCLOIDEA (CYC), and PROLIFERATING CELL NUCLEAR ANTIGEN FACTOR 1 and 2 (PCFs); hence, it was termed the TCP family (Cubas et al., 1999; Kosugi et al., 2015; Luo et al., 1996; Doebley et al., 1997). TCP genes are classified into two classes based on differences within the TCP domain: class I (Kosugi and Ohashi, 2002), also termed the PCF class (Cubas, 2002) or TCP-P class (Navaud et al., 2007), and class II, also known as the TCP-C class. Class II can be further subdivided into two clades based on differences in the TCP domain: the CIN clade, which is involved in lateral organ development, and the CYC/TB1 clade (or ECE clade) (Howarth and Donoghue, 2006), which is mainly involved in the development of axillary meristems giving rise to either flowers or lateral shoots (Cubas and Martı, 2009; Nicolas and Cubas, 2015; Li et al., 2019; Li et al., 2021).

*BRANCHED 1* (*BRC1* or *TCP18*) encodes a member of the TCP family and is an integrator of multiple internal and external signals that act in the axillary buds to suppress shoot branching. For example, in *Arabidopsis*, *BRC1* is expressed in developing buds to arrest bud development, and downregulation of *BRC1* leads to branch outgrowth (Aguilar-Martínez et al., 2007; Finlayson, 2007). In tomato, two *BRC1-like* paralogs, *SlBRC1a* and *SlBRC1b*, are expressed in arrested axillary buds, with *SlBRC1a* transcribed at much lower levels than *SlBRC1b* (Martín-Trillo et al., 2011). Some researchers have reported that *BRC1* plays a role in controlling branching in crop brassicas such as *Brassica napus* (AACC, 2*n* = 38) (Li et al., 2020) and *Brassica juncea* (AABB, 2*n* = 36) (Muntha et al., 2019), indicating that BRC1 may have conserved functions in regulating the growth and development of axillary buds in crop brassicas.Many genes are involved in BRC1-mediated axillary bud growth (Liu et al., 2017; Shen et al., 2019; Xie et al., 2020), indicating that *BRC1* as integrator in controlling shoot branching is regulated by multiple transcription factors.

*BRC1* is also reported to participate in the regulation of flowering time. For example, BRC1 can interact with FT and TSF (but not with TFL1) and modulates florigen activity in the axillary buds to prevent premature floral transition of the apical meristems (Niwa et al., 2013). Certain class II TCP proteins containing BRC1 can interact with FD, raising the possibility that they may be incorporated into the FT–FD module to control photoperiodic flowering (Li et al., 2019). In hybrid aspen, BRC1 physically interacts with FT2, further reinforcing the effect of a short photoperiod and triggering growth cessation by antagonizing FT action (Maurya et al., 2020). The allotetraploid *Brassica juncea*, which originated from *Brassica rapa* (AA, 2*n* = 20) and *Brassica nigra* (BB, 2*n* = 16) by natural interspecific hybridization, is an economically important crop plant in China. Five *BRC1* homologous genes with a conserved TCP domain are present in the genome of *B. juncea* based on information in the BRAD database; two homologs (BjuVA03G39990 and BjuVA01G37280) originated from *B. rapa*, whereas the other three homologs (BjuVB07G28850, BjuVB01G36290, and BjuVB07G09230) originated from *B. nigra*. It is not clear whether the BjuBRC1 homologs function in the regulation of flowering in *B. juncea*.

In this study, we isolated four *BjuBRC1* genes from *B. juncea* and selected one (*BjuBRC1*-1, BjuVB07G09230) for study in detail. In *B. juncea*, *BjuBRC1*-1 expression was induced by short days (SD), and its heterologous overexpression in *Arabidopsis* delayed flowering time under LD. Four BjuBRC1 proteins were capable of interaction with BjuFT, whereas only BjuBRC1-1 could directly bind to the promoter of *BjuFUL* to repress its expression. These mechanisms indicated that BjuBRC1-1 may function in the regulation of flowering.

## Materials and methods

### Plant materials and growth conditions

The *Brassica juncea* plant materials used in this study were the homozygous line mustard “J92” which was provided by our laboratory. *B. juncea* seedlings were grown in the artificial climate chamber under long-day conditions (22°C 16-h light/20°C 8-h dark).

Wild-type *Arabidopsis* (Columbia ecotype) and its *brc1* mutant (T-DNA insert lines; results of positive detection by PCR amplification are shown in **Supplementary Figure 1**) used in this study were obtained from ABRC (https://www.arashare.cn/index/Product/index). After surface sterilization, all *Arabidopsis* seeds were sown on half-strength MS media plates supplemented with 3% sucrose to allow seed germination and seedling growth, and the plates were placed in the artificial climate chamber (22°C 16-h light/20°C 8-h dark). Then, *Arabidopsis* were transferred from plates to soil after about 5 days for growth.

The seeds of *Nicotiana benthamiana* were directly sown into the soil and were grown in the chamber for 1 month (25°C, 16-h light/8-h dark).

### Multiple-sequence alignment and phylogenetic analysis

The coding sequence of *Arabidopsis thaliana AtBRC1* was downloaded from The Arabidopsis Information Resource (https://www.arabidopsis.org/) and was used as the query for a BLAST search of BRAD to obtain *BRC1* sequences in *Brassica* species. The full-length amino acid sequences of BjuBRC1 proteins (BjuVA03G39990, BjuVA01G37280, BjuVB07G28850, BjuVB01G36290, and BjuVB07G09230) were downloaded from the Brassicaceae Database (BRAD, http://39.100.233.196/). In addition, all amino acid sequences for BRC1 proteins of other *Brassica* species were obtained from BRAD, comprising *B. rapa* (BraA01g035600.3.5C and BraA03g038870.3.5C), *B. nigra* (BniB035325, BniB039293, and BniB017427), *B. oleracea* (BolC1t04530H, BolC5t33435H, and BolC3t17248H), *B. napus* (BnaDarC03p49500.1, BnaDARA03p40710.1, BnaDARC05p47080.1, BnaDARA01p35910.1, and BnaDARC01p45680.1), and *B. carinata* (BcaC01g03693, BcaB04g18990, BcaB04g18989, BcaB06g25899, BcaC05g28604, and BcaC09g48810). A multiple-sequence alignment was generated with BioXM2.7 software. Phylogenetic analysis was performed with the neighbor-joining method with 1,000 bootstrap replicates using MEGA10.2.0. The phylogenetic tree for the BRC1 proteins was visualized and adjusted with EvolView (https://www.evolgenius.info/evolview/).

### RNA extraction, reverse transcription, and gene cloning

Leaves and flowers were sampled from 5-week-old *B. juncea* plants and were immediately frozen in liquid nitrogen. Total RNA was extracted from the samples using the Biospin Plant Total RNA Extraction Kit (BioFlux) following the manufacturer’s instructions. Genomic DNA (gDNA) was digested in a 15-μl reaction mixture containing 3 μl 5× gDNA Digester Mix, 6 μl total RNA, and 6 μl RNase-free H_2_O, at 42°C for 2 min). The purified RNA was reverse-transcribed in a 20-μl reaction volume using the Hifair III 1st Strand cDNA Synthesis SuperMix Kit (YEASEN) in accordance with the manufacturer’s instructions. The reverse transcription thermal profile comprised 25°C for 5 min, followed by 55°C for 15 min and 85°C for 5 min. All cDNA samples were diluted with ddH_2_O (1:4, v/v). The diluted cDNA was used directly for PCR amplification.

Based on the conserved sequences in the *Brassica BRC1* genes, gene-specific primers (BRC1-F1, BRC1-F2, and BRC1-R1) were designed to amplify the *BjuBRC1* genes in this study. Based on sequences of *BjuBRC1*-1, four truncated genes were subcloned by special primers. In addition, two *BjuFT* genes (BjuVB05G49700 and BjuVA07G33060) were cloned using the same procedure described above. All primers used are listed in **Supplementary Table 1**.

### Subcellular localization assay

The *BjuBRC1*-1 coding region without the stop codon (and two truncated sequences *BjuBRC1*-1-I and *BjuBRC1*-1-II, respectively) was subcloned into the pCAMBIA1300-*GFP* vector *via* double-enzyme digestion with *Xba*I and *Kpn*I. The recombinant plasmid p*35S*::*BjuBRC1*-1*-GFP* and the empty vector pCAMBIA1300-*GFP* were transformed separately into *Agrobacterium tumefaciens* strain GV3101. The leaves of 1-month-old *Nicotiana benthamiana* plants were infiltrated with *A. tumefaciens* cells harboring pCAMBIA1300-*GFP*, pCAMBIA1300-*BjuBRC1*-1*-GFP*, or the corresponding construct containing a truncated *BjuBRC1*-1 sequence. The *N. benthamiana* leaves infiltrated with *A. tumefaciens* were incubated in the dark for 1 day and then transferred to a growth chamber maintained at 25°C with a 16-h/8-h (light/dark) photoperiod for 24–48 h. The leaves were used for visualization of the GFP signal with a laser scanning confocal microscope (LSM78010800, ZEISS).

### Expression analysis

*Brassica juncea* plants used for expression analysis were grown in an artificial climate chamber under LD (22°C 16-h light/20°C 8-h dark) or SD (22°C 8-h light/20°C 16-h dark). At least three leaf (stems, flowering buds, and flowerings) samples from each day-length treatment were collected for subsequent analysis. All *Arabidopsis* plants were grown in the artificial climate chamber under LD, and their 2-week-old leaves were sampled.

Total RNA extraction and reverse transcription were conducted as described under “RNA extraction, reverse transcription and gene cloning”. The cDNA was used either immediately in the following reaction or stored at −80°C until use. Quantitative real-time PCR (qRT-PCR) analysis was performed using the SYBR qPCR SuperMix Plus (Novoprotein Scientific) on a CFX96™ Real-Time PCR Detection System (Bio-Rad). The primers used for qPCR analysis of *BjuBRC1*-1 (BRC1-qPCR-F and BRC1-qPCR-R) and *AtFUL* (FUL-qPCR-F and FUL-qPCR-R) are listed in **Supplementary Table 1**. The *BjuACTIN2* and *AtTUB2* genes were used for normalization of the *B. juncea* and *Arabidopsis* samples, respectively. Each qPCR analysis was performed in a volume of 20 μl containing 10 μl 2× NovoStart^®^ SYBR qPCR SuperMix Plus, 1 μl cDNA, 2 μl of the primer pair, and 7 μl ddH_2_O. The qPCR protocol incorporated “two-step amplification” for greater specificity as follows: 95°C for 1 min, 95°C for 20 s, and 60°C for 1 min, conducted for a maximum of 40 cycles. The relative expression level was calculated using the 2^−ΔΔ^ *^C^* ^t^ method (Livak and Schmittgen, 2001). Each sample was quantified in triplicate with three biological replicates. GraphPad Prism 5 software was used to graphically visualize the results. The significance of differences among the data was analyzed using Student’s *t-*test with SPSS software (18.0). The primers used are listed in **Supplementary Table 1**.

### Overexpression vector construction and plant transformation

*BjuBRC1*-1 (full-length) was subcloned and inserted into the pCAMBIA1300 vector containing the *HYG* resistance gene under the control of the *35S* promoter. The recombination plasmid was introduced into *A. tumefaciens* strain GV3101 using the freeze–thaw method and transformed into *Arabidopsis* by the floral dip method (Bent and Clough, 1998). First, hygromycin was used to screen positive transgenic plants, and then PCR amplification was performed to verify the presence of the transgene. Three T_1_ transgenic *Arabidopsis* lines and their T_3_ progeny were subjected to qPCR for gene expression analysis.

### DNA extraction and promoter cloning

The gDNA used for cloning of promoters was extracted using the Plant Genomic DNA Kit (TIANGEN) from young leaves of *B. juncea* following the manufacturer’s instructions. The gDNA product was stored at −80°C until use. The primers used for PCR amplification of the promoter region (the 1–2-kb region upstream of the start codon “ATG”) were designed based on the reference sequences (*ProBjuFUL*, BjuVA03G46090, 1,200 bp; *ProBjuFT*, BjuVB05G49700, 2,000 bp; and *ProBjuSOC1*, BjuVB08G24640, 800 bp) accessed from BRAD. All primers used are listed in **Supplementary Table 1**. Moreover, *ProBjuSOC1* (pAbAi-*ProBjuSOC1* vector) was provided by our laboratory.

### Yeast two-hybrid assay

To detect protein–protein interactions using a yeast two-hybrid system, four *BjuBRC1* homologous genes and *BjuFT* were inserted into the pGADT7 and pGBDT7 vectors, respectively. The recombinant plasmids pGBDT7-*BjuBRC1s* and pGBDT7-*BjuFT* were separately transformed into yeast strain Y2H Gold, whereas the pGADT7-*BjuBRC1s* and pGADT7-*BjuFT* plasmids were transformed into yeast strain Y187. Each prey (pGADT7) yeast strain was fused with the bait (pGBDT7) yeast strain with 2× YPDA liquid medium, and the recombined strains were cultured on DDO (SD/−Leu/−Trp) plates for 3 days at 30°C. Three independent colonies on the DDO plates were chosen to detect the interactions on QDO (SD/−Leu/−Trp/−Ade/−His) plates supplemented with X-α-gal (40 µg/ml) and aureobasidin A (AbA; 0.3 µg/ml).

### Bimolecular fluorescent complimentary assays

The coding sequences of four *BjuBRC1* genes and *BjuFT* were subcloned into the pVYCE and pVYNE vectors with the specific primers, respectively. *Agrobacterium* solutions containing pVYNE and pVYNE–*BjuBRC1s* were respectively mixed with the same volumes of *Agrobacterium* solutions containing pVYCE–*BjuFT*. Similarly, different combinations were held to detect the interactions among four BjuBRC1 proteins. The following procedures were similar to those used in the above subcellular localization experiment, and the fluorescent was detected by LSCM.

### Luciferase complementation assay

The full-length *BjuBRC1*-1 and *BjuFT* were separately cloned into the pCAMBIA1300-CLuc (pC-C) and pCAMBIA1300-NLuc (pC-N) vectors, respectively (Zhou et al., 2018). *Agrobacterium* suspensions harboring pC-C and pC-C–*BjuBRC1*-1 were respectively mixed with the same volumes of *Agrobacterium* suspensions harboring pC-N–*BjuFT*.

Before bacterial infiltration, healthy and vigorous *N. benthamiana* plants were selected and placed under light for more than 1 h to ensure that most stomata were open and suitable for agroinfiltration. Small leaves were not suitable for agroinfiltration. The negative controls and tested protein pairs (pC-C–*BjuBRC1* and pC-N–*BjuFT*) were infiltrated into the same leaf to avoid false positives and negatives caused by differences in physiological conditions between leaves. The infiltrated plants were incubated in a greenhouse for 40–48 h before measurement of luminescence. Then, a 1-mM D-luciferin solution was sprayed onto the leaves of *N. benthamiana*, ensuring that the leaves were completely wet, and then the plant was placed in the dark for 5–8 min to allow the chlorophyll luminescence to decay. Luminescence images were captured using a IVIS Imaging System.

### Yeast one-hybrid assay

Four *BjuBRC1* genes (and four truncated *BjuBRC1*-1) were respectively inserted into the pGADT7 vector by double-enzyme digestion with *NdeI and EcoR I*. The promoters of *BjuFUL*, *BjuFT*, and *BjuSOC1* were cloned from leaves of *B. juncea* ‘J92’ and then recombined separately into the pAbAi vector. Using the Matchmaker Gold Yeast One-Hybrid Library Screening System (TaKaRa), the linearized plasmids pAbAi-*ProBjuFUL*, pAbAi-*ProBjuFT*, and pAbAi-*ProBjuSOC1* were introduced into the yeast strain Y1H Gold and cultured on SD/−Ura agar plates. The minimum concentration of AbA (0–800 ng/ml) that inhibited yeast growth was screened on SD/−Ura/AbA plates. Subsequently, the pGADT7-*BjuBRC1* plasmids were transformed into yeast strain Y1H Gold harboring pAbAi-*ProBjuFT*, pAbAi-*ProBjuFUL*, or pAbAi-*ProBjuSOC1*. The yeast strains were successively plated on SD/−Leu and SD/−Leu/AbA media. The growth of the yeast strains at 30°C was assessed for testing the protein–DNA interactions.

### Dual-luciferase reporter system

The *BjuFUL* promoter containing the W-box element was amplified and digested with *Hind*III and *Xho* I and then recombined into the pGreenII0800-LUC vector as the reporter plasmid (Hellens et al., 2005). Similarly, *BjuBRC1* was digested with *Sac*I and *BamH* I and then inserted into the pGreenII62-SK vector as the effector plasmid. Using the freeze–thaw method, both pGreenII62-0800-*ProBjuFUL* and pGreenII62-SK-*BjuBRC1* plasmids were introduced into *A*. *tumefaciens* strain GV3101 and then cultured at 28°C until OD_600_ = 0.9–1.1 in 50 ml YEB medium supplemented with 50 μg/ml kanamycin and 25 μg/ml rifampicin. The cells were harvested and resuspended in suspension medium (10 mM MgCl_2_, 100 μl of 100 mM acetosyringone, and 100 μl of 0.5 M MES) and then cultured for 2–3 h at room temperature in the dark. The agrobacteria were infiltrated into 1-month-old *N. benthamiana* leaves, and the infiltrated plant was placed in the dark for 24 h and then transferred to the light for 36 h. Luciferase signals were measured with a microplate reader. Transcriptional activity in the infiltrated tobacco leaves was determined as the ratio of LUC to REN with the Dual Luciferase Reporter Gene Assay Kit (YEASEN).

### Luciferase activity assay

*Nicotiana benthamiana* leaves were injected with *ProBjuFUL*::*LUC* or co-injected with *35S*::*BjuBRC1* and *ProBjuFUL*::*LUC* and incubated at 22°C for 2–3 days. The injected leaves were detached and sprayed with 1 mM of D-luciferin solution (potassium salt, APExBIO) and incubated in the dark for 5–7 min. Luciferase luminescence in the infiltrated area was visualized using an IVIS Lumina Series III10899 Imaging System (America, PerkinElmer).

## Results

### Gene cloning and characterization of *BjuBRC1* homologs in *B. juncea*

**Figure 1:**
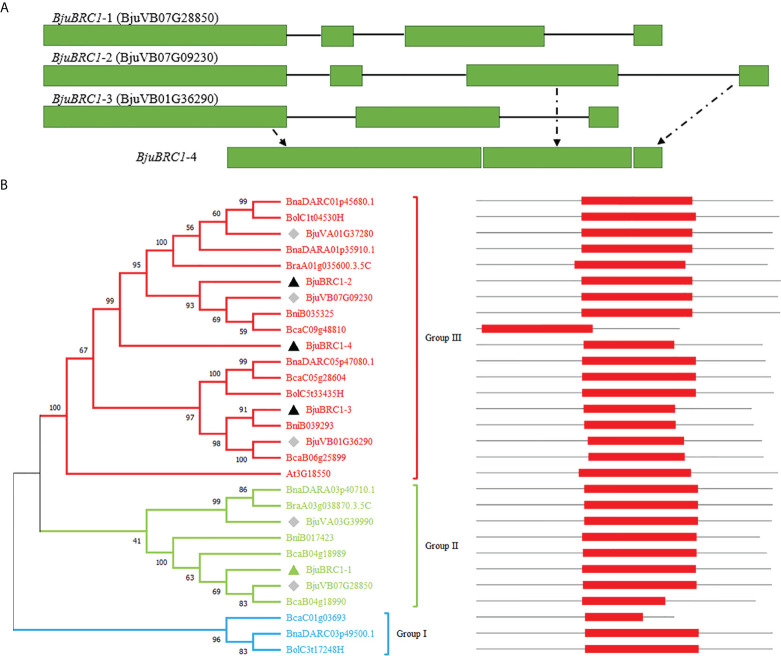
*BjuBRC1* gene structure and phylogenetic tree. **(A)** *BjuBRC1* gene structure. The green rectangle parts indicate exons; the black lines indicate introns. *BjuBRC1*-4 has no accession number in the BRAD database, whose gene structure consists of parts of *BjuBRC1*-2 and *BjuBRC1*-3 (drawn by dotted lines). **(B)** Phylogenetic tree of BRC1 proteins from different plants in *Brassicas*; the accession number of all genes are shown in the picture. BRC1 proteins are classed as three groups (red, green, and blue) according to their evolutionary relationship. Triangles indicate the genes cloned in this study, and the green triangle indicates representative genes chosen to be studied. The diamonds indicate homologous genes of *BjuBRC1* in BRAD. The location of TCP domains (red rectangle) in AA sequences are shown on the right.

In this study, *BjuBRC1* genes were cloned from *B. juncea* by PCR using gene-specific primers. Four homologous *BjuBRC1* genes in the genome of *B. juncea* were sequenced, and the respective gene structures were analyzed (**Figure 1A**). The results indicated that their gene sequences were significantly different. Notably, the N-terminal region of the *BjuBRC1*-4 sequence was highly similar to that of *BjuBRC1*-3, whereas the central and C-terminal regions of *BjuBRC1*-4 were similar to those of *BjuBRC1*-2 (**Figure 1A**, **Supplementary Figure 2**). Therefore, *BjuBRC1*-4 was indicated to be a unique homolog that was not yet registered in the BRAD database.

A multiple alignment of the full-length amino acid sequences was generated to examine the phylogenetic relationships among the BjuBRC1 proteins and their orthologs from multiple species of *Brassica*. A phylogenetic tree was constructed using the neighbor-joining algorithm (**Figure 1B**). Phylogenetic analysis revealed the evolutionary relationship of BRC1 homologs in *Brassicas*. The Pfam database (http://pfam.xfam.o-rg/) was used to predict the conserved domains in the BRC1 protein sequences. The BjuBRC1-1 protein showed the highest similarity to the AtBRC1 (At3G18550) sequence, based on a BLAST search of BRAD. Therefore, BjuBRC1-1 was selected as representative to analyze the function of *BjuBRC1* genes.

### Expression patterns of *BjuBRC1*-1 in *B. juncea*

The expression patterns of *BjuBRC1*-1 at different developmental stages (in leaves) and in diverse organs (under LD, sampled while flowering) were analyzed by qPCR. The expression levels in the flower, floral bud, and leaf were higher than those in the root and stem (**Supplementary Figure 3A**).

It has been reported that *BRC1* is induced under SD, resulting in growth cessation, in hybrid aspen (Maurya et al., 2020). Therefore, we explored the relative expression levels of *BjuBRC1*-1 in *B. juncea* leaves under LD and SD by qPCR. The results revealed that *BjuBRC1*-1 was expressed more abundantly under SD than under LD. However, the expression level decreased gradually with growth of the plant (**Supplementary Figure 3B**). Furthermore, SD delayed the growth and flowering of *B. juncea* plants (**Supplementary Figure 4**). Thus, *BjuBRC1*-1 may negatively regulate flowering in *B. juncea*.

### Subcellular localization

**Figure 2:**
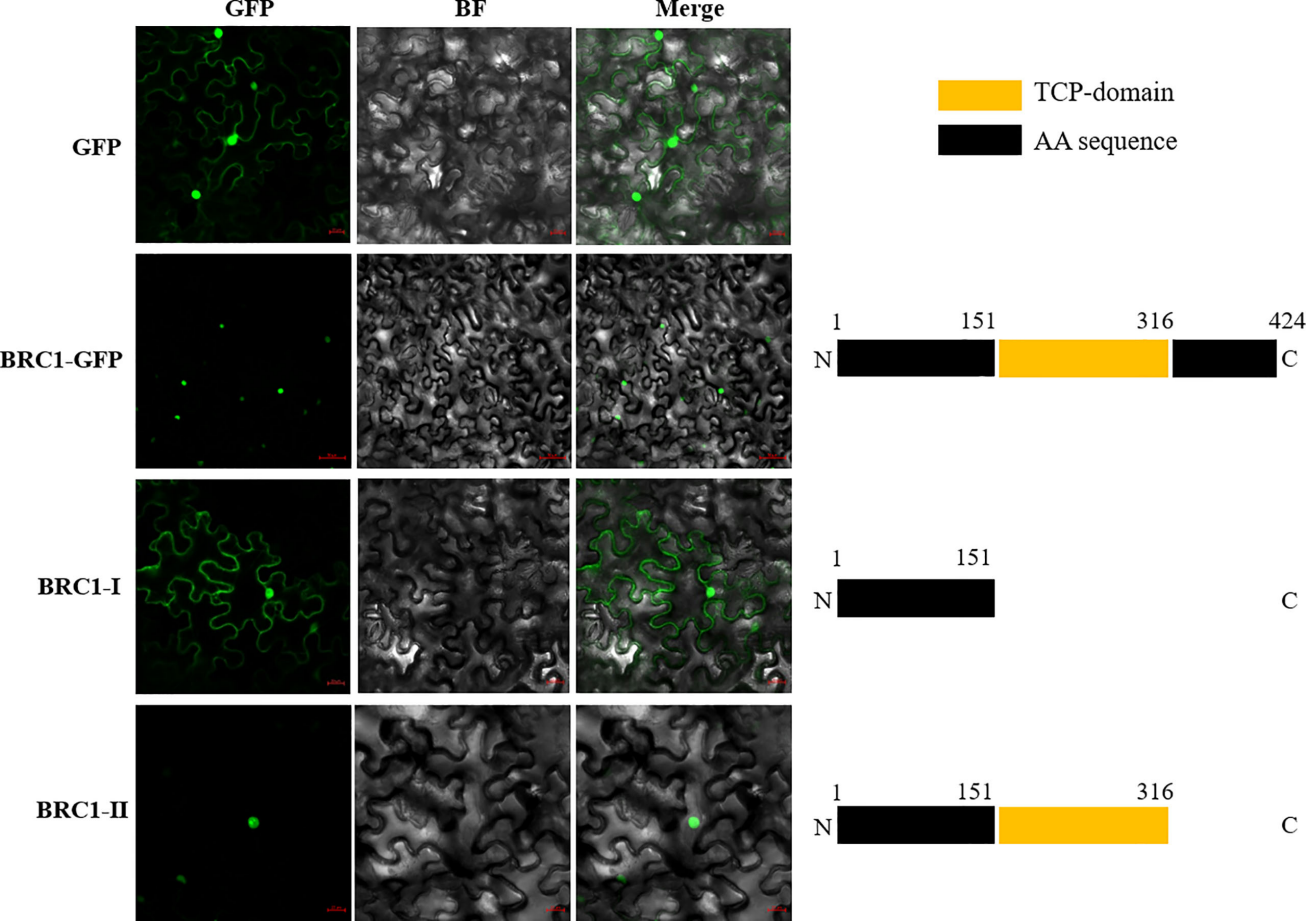
The subcellular localization of BjuBRC1-1 protein and its two truncated proteins. The schematic diagram of two truncated proteins is shown on the right. GFP, GFP fluorescent; BF, bright-field; Merged, merged image of GFP and BF.

It was speculated that the BjuBRC1 protein, as a transcription factor, would be localized in the nucleus. To investigate the subcellular localization of BjuBRC1-1, we fused *BjuBRC1*-1 to the N-terminus of the green fluorescent protein gene (*GFP*) and used *Agrobacterium*-mediated transformation to transiently express the fusion protein in leaves of *Nicotiana benthamiana*. The fluorescent signal of the BjuBRC1-1-GFP protein was only observed in the nucleus, whereas the GFP control signal was observed in the cytoplasm and nucleus (**Figure 2**). These results indicated that BjuBRC1-1 was a nuclear-localized protein and acted as a transcription factor.

In potato, alternative splicing leads to the generation of two different protein isoforms from StBRC1a, namely, StBRC1a^L^ (with a strong activation domain in the C-terminal region) and StBRC1a^S^ (which lacks an activation domain in the C-terminal region). StBRC1a^L^ is a nuclear protein and acts as a strong transcription factor that causes growth arrest, whereas StBRC1a^S^ is localized to the cytoplasm and acts as a dominant-negative factor that antagonizes StBRC1a^L^, mainly by sequestering it outside the nucleus (Nicolas et al., 2015). Therefore, we constructed two truncated proteins, BjuBRC1-1-I and BjuBRC1-1-II (the structure of the truncated proteins is shown in **Figure 2**), to examine their subcellular localization to assess whether a similar molecular mechanism may exist in *B. juncea*. BjuBRC1-1-I was localized in the cytoplasm and nucleus, whereas BjuBRC1-1-II was detected in the nucleus only (**Figure 2**), indicating that the mechanism in *B. juncea* may differ from that of potato.

### Overexpression of *BjuBRC1*-1 in *Arabidopsis* repressed flowering

**Figure 3:**
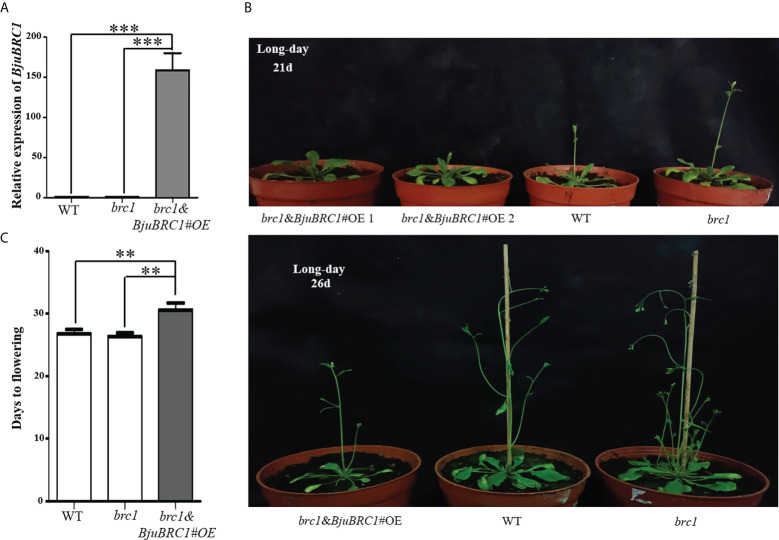
Flowering time comparison of distinct Arabidopsis lines. **(A)** The relative expression level of *BjuBRC1*-1 in transgenic *Arabidopsis* (*brc1* mutant background), WT, and *brc1 Arabidopsis*. “***” indicates significant difference, p < 0.001, by Student’ s t-test. **(B)** Overexpression of *BjuBRC1*-1 delayed flowering. **(C)** Days to flowering of the WT, *brc1*, and the transgenic *Arabidopsis*. Error bars represent SE. “**” indicates significant difference, p < 0.01, by Student’ s t-test.

To explore the biological functions of *BjuBRC1*, an overexpression vector driven by a *35S* promoter was constructed. After *Agrobacterium* infection of *Arabidopsis* flowers (Bent and Clough, 1998), we obtained transgenic *Arabidopsis* plants in the *brc1* mutant background. The transgenic *Arabidopsis* lines were verified by PCR (at the DNA level) and qPCR (at the RNA level) (**Figure 3A**, **Supplementary Figure 5**). Three transgenic lines were obtained, and their T_3_ progeny was selected for further phenotypic analysis. No difference was observed in the expression level of *BjuBRC1* between transgenic *Arabidopsis* line 3 and the wild type (WT; *brc1* mutant) in T_3_ plants; therefore, line 3 was not included in the statistics. Compared with WT plants, two transgenic lines (lines 1 and 2) showed a late-flowering phenotype, whereas no significant difference in flowering time was observed between the *brc1* mutant and WT (**Figure 3B**). The indication that *BjuBRC1* impacted on flowering time was consistent with the influence of BRC1 in hybrid aspen and *Arabidopsis* (Niwa et al., 2013; Maurya et al., 2020). In addition, the transgenic lines developed similar branches with the *brc1* mutant (**Supplementary Figure 6**), which indicated that *BjuBRC1*-1 may not play a role in the regulation of branching.

### BjuBRC1 homologs interacted with BjuFT *in vivo*

**Figure 4:**
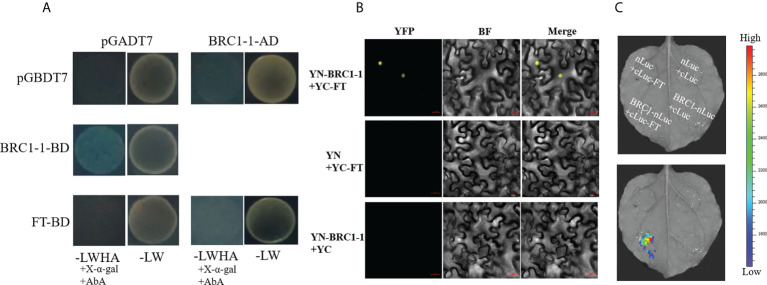
Interaction analysis between BjuBRC1-1 and BjuFT. **(A)** Yeast two-hybrid assay between BjuBRC1-1 and BjuFT. **(B)** BiFC assays between YN-BjuBRC1-1 and YC-BjuFT. YFP, YFP fluorescence; BF, bright-field; Merged, merged image of YFP and BF **(C)** LCI assays of BjuBRC1-nLuc with cLuc-BjuFT.

It has been reported that BRC1 physically interacts with FT and inhibits flowering in axillary buds by antagonizing FT action in *Arabidopsis* (Niwa et al., 2013) and hybrid aspen (Maurya et al., 2020). Five *FT* homologous genes were detected in the genome of *B. juncea* (BjuVB05G49700, BjuVA02G18040, BjuVB03G38700, BjuVA07G33060, and BjuVA07G42420) through BRAD. In this study, two homologous *BjuFT* genes (BjuVB05G49700 and BjuVA07G33060) that showed high sequence similarities were cloned, and one (BjuVB05G49700) was selected for the following analysis. A yeast two-hybrid assay was performed to examine the possible interactions between BjuFT and four BjuBRC1 proteins. All fused yeast strains were unable to grow on the QDO medium supplemented with AbA and X-α-gal (**Figure 4A**). Interaction of BjuBRC1-1 with FT was not detected in yeast. To further explore the interaction between BjuBRC1-1 and BjuFT, BiFC and LCI assays were performed. The results revealed that BjuBRC1-1 was capable of interacting with BjuFT *in vivo* (**Figures 4B, C**).

To further assess the conservation of the interaction between BRC1 and FT in *B. juncea*, the interactions of three homologous BjuBRC1 proteins with BjuFT were studied. A yeast two-hybrid assay was conducted to detect the separate interactions between the BjuBRC1 proteins and BjuFT. The results showed that interactions of the three BjuBRC1 homologs (BRC1-2, BRC1-3, and BRC1-4) with BjuFT were not detected in yeast (**Supplementary Figure 7**).

**Figure 5:**
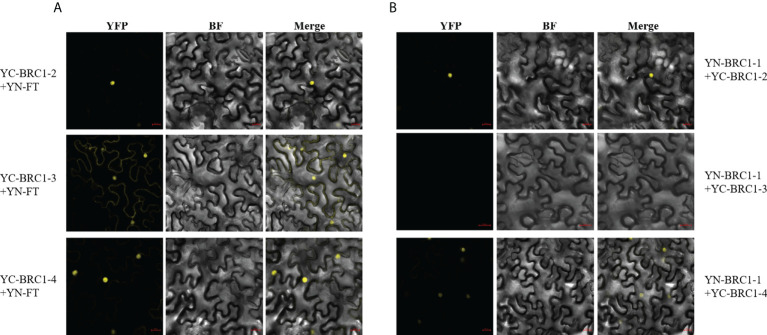
Detections of three BjuBRC1 homologous proteins interact with BjuFT or each other in vivo. **(A)** BiFC assays between three BjuBRC1 proteins and BjuFT **(B)** BiFC assays between BjuBRC1-1 and BjuBRC1-2, BjuBRC1-3, or BjuBRC1-4. YFP, YFP fluorescence; BF, bright-field; Merged, merged image of YFP and BF.

Next, BiFC assays were performed and the results indicated that BjuFT was capable of interacting with BjuBRC1-2, BjuBRC1-3, and BjuBRC1-4 *in vivo* (**Figure 5A**, **Supplementary Figure 8**). Notably, BjuBRC1-3 was colocalized in the nucleus and cytomembrane and interacted with BjuFT, which was distinct from the other BjuBRC1 homologs. These results suggested that BjuBRC1 may regulate flowering by interacting with BjuFT to antagonize the CO/FT pathway, resulting in downregulation of its downstream targets (Niwa et al., 2013; Maurya et al., 2020).

### BjuBRC1 homologs formed heterodimers

BRC1 forms homo- and heterodimers in *Solanum tuberosum* (Nicolas et al., 2015). To explore if a similar mechanism may operate in *B. juncea*, the interactions of BjuBRC1 proteins were examined by means of a yeast two-hybrid assay. The results indicated that at least BjuBRC1-3 could not form heterodimers with itself and other homologous proteins in yeast (**Supplementary Figure 7B**). A BiFC assay in *N. benthamiana* leaves indicated that BjuBRC1-1 could form heterodimers with BjuBRC1-2 or BjuBRC1-4, but not with BjuBRC1-3, which was consistent with the results of the yeast two-hybrid assay (**Figure 5B**, **Supplementary Figure 9**). In addition, we further tested other BiFC combinations to detect homologous interactions and the results showed there were no YFP signals in these BiFC combinations (**Supplementary Figure 10**). These results provided evidence that BjuBRC1 proteins could form heterodimers, but whether this mechanism mediates the regulation of flowering requires further study.

### BjuBRC1-1 repressed *BjuFUL* expression

**Figure 6:**
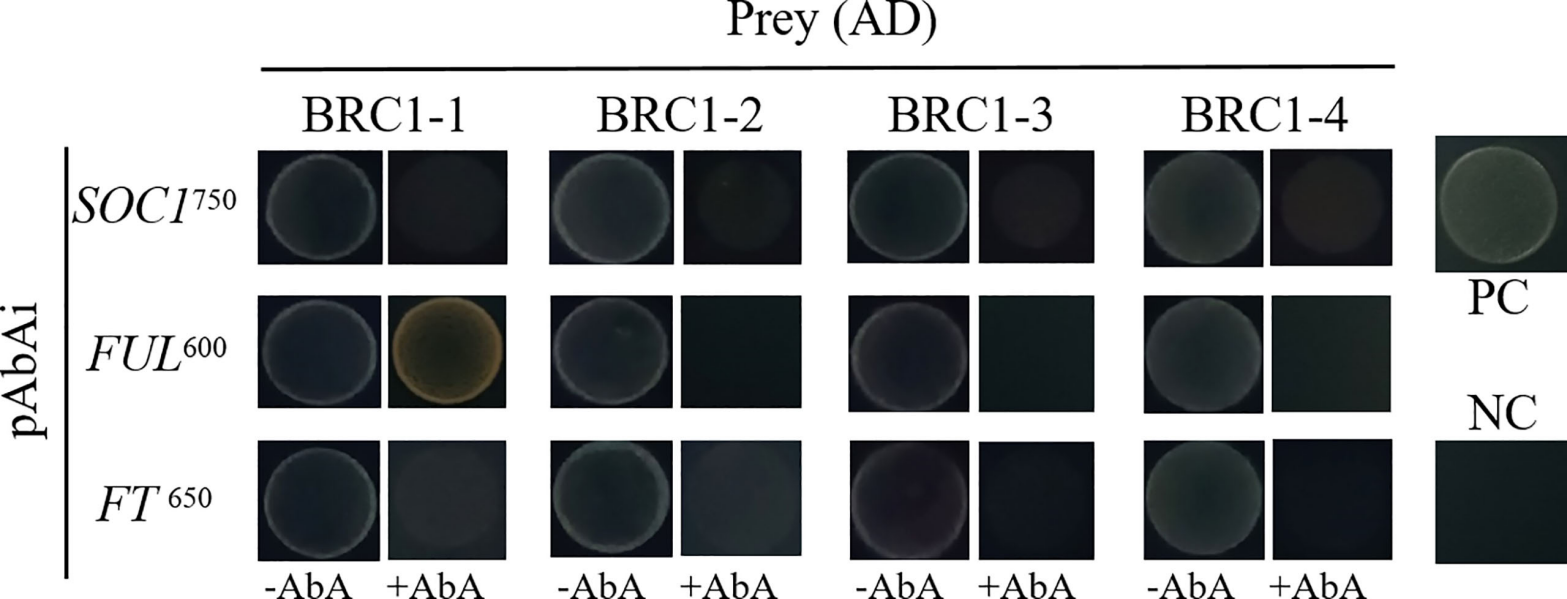
Yeast one hybrid assay. Yeast one-hybrid assay to test whether the four BjuBRC1 proteins could directly bind to the promoters of *BjuFT*, *BjuFUL*, or *BjuSOC1*. pGADT7-*BjuBRC1s* was transformed into yeast Y1H strains in combination with pAbAi-*ProBjuFT*, pAbAi-*ProBjuFUL*, and pAbAi-*ProBjuSOC1*, respectively. The transformed strains all were grown on SD/-Leu selective media containing aureobasidin A (ABA) (650, 600, and 750 μg/l, respectively). PC, positive control; NC, negative control.

To examine if BjuBRC1 proteins may influence the flowering time by directly binding to the promoters of floral regulatory genes in *B. juncea*, we performed a yeast one-hybrid assay to test the interactions of BjuBRC1s with *ProBjuFUL* (BjuVA03G46090, one of the six detected in BRAD), *ProBjuFT* (BjuVB05G49700, one of the five in BRAD), and *ProBjuSOC1* (BjuVB08G24640, one of the six in BRAD). Only the yeast strain harboring *ProBjuFUL* and pGADT7-*BjuBRC1*-1 was able to grow on SD/−Leu/AbA^600^ plates, indicating that BjuBRC1-1 may bind to the promoter of *BjuFUL* (**Figure 6**). The other BjuBRC1 homologous proteins were not capable of binding to the promoter of *BjuFUL*, which indicated that the mechanism of BjuBRC1 binding to *ProBjuFUL* was not conserved (**Figure 6**).

**Figure 7:**
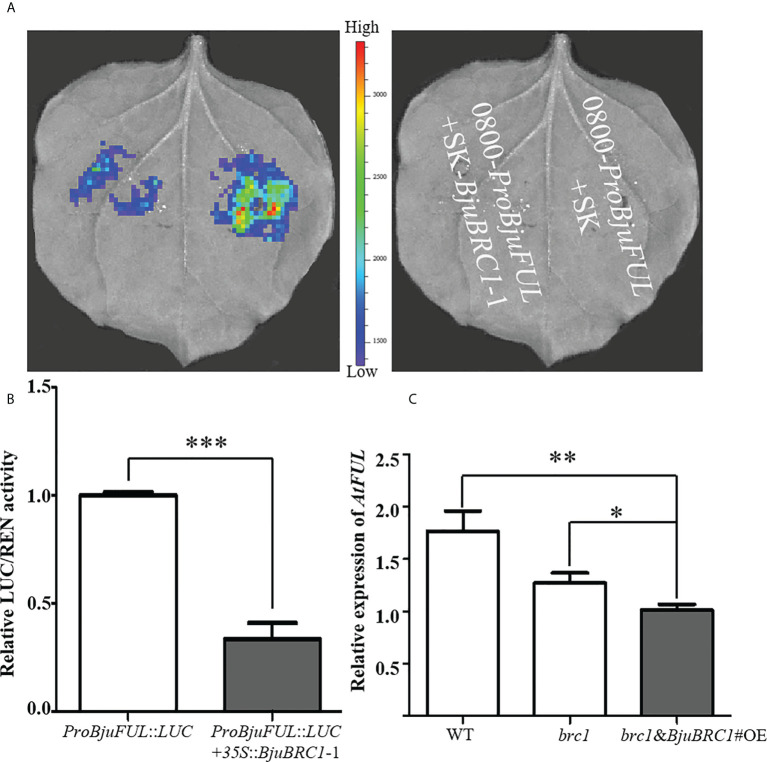
BjuBRC1-1 regulates the expression of *BjuFUL*. **(A)** LUC assays to detect the activation/repression function of BjuBRC1-1 protein to *ProBjuFUL* **(B)** Dual-luciferase reporter assay to verify whether BjuBRC1-1 protein regulates the transcription of *BjuFUL.* **(C)** Relative expression levels of *AtFUL* in transgenic *Arabidopsis* compared with WT or *brc1* mutant. Error bars represent SE. “***” indicates significant difference, p < 0.001; “**”, p < 0.01; “*”, p < 0.05. By Student’ s t-test.

To explore the amino acid residues required for binding to *ProBjuFUL*, four truncated proteins of BjuBRC1-1 were generated (**Supplementary Figure 11A**). Yeast one-hybrid assays demonstrated that only the full-length BjuBRC1-1 protein was capable of interacting with the *BjuFUL* promoter (**Figures 6**, **Supplementary Figure 11**). Subsequently, a LUC assay and dual-luciferase reporter system were performed to clarify the mechanism by which BjuBRC1-1 regulated the downstream gene *BjuFUL*. The results indicated that BjuBRC1-1 was able to repress the transcription of *BjuFUL* (**Figures 7A, B**).

To verify this conclusion, a qPCR analysis was performed to quantify the expression level of *AtFUL* in leaves of WT, *brc1* mutant, and transgenic *Arabidopsis* plants. In accordance with the abovementioned results, *AtFUL* expression was reduced in transgenic *Arabidopsis* compared with that in the WT and *brc1* mutant (**Figure 7C**).

Collectively, from the present results, we concluded that BjuBRC1-1 was capable of directly binding to the promoter of *BjuFUL* to repress its expression in *B. juncea* and speculated that this mechanism may be involved in the regulation of flowering.

## Discussion

Considerable efforts have been made to illuminate the complex molecular networks that regulate flowering time owing to its importance for reproductive success and crop yields. The BRC1 proteins, a member of TCP transcriptional factors family, are well known to function as an essential hub for different signals to regulate bud growth in many plant species (Aguilar-Martínez et al., 2007; Dun et al., 2009; Leyser, 2009; Beveridge and Kyozuka, 2010; Rameau et al., 2015; Nicolas and Cubas, 2016; Xie et al., 2020). In addition, *BRC1* is involved in the regulation of flowering in certain plants, such as *Arabidopsis* and hybrid aspen (Niwa et al., 2013; Maurya et al., 2020). However, the functions of *BRC1* genes in Brassica species, especially *B. juncea*, remain unknown.

In this study, we identified four *BRC1* genes in the *B. juncea* genome and one (*BjuBRC1*-1) was selected for study in detail as a representative of the genes. A subcellular localization analysis indicated that BjuBRC1-1 was functional as a transcription factor localized to the nucleus (**Figure 2**). A qPCR analysis demonstrated that *BjuBRC1*-1 could be induced by SD rather than LD (**Supplementary Figure 3B**), which was consistent with the induction of *BRC1* in hybrid aspen (Maurya et al., 2020). Short days is a negative factor in the regulation of flowering plant development; therefore, we speculated that *BjuBRC1*-1 may function as a repressor of flowering.

To evaluate this hypothesis, we generated three transgenic *Arabidopsis* lines (in the *brc1* mutant background) that ectopically expressed *BjuBRC1*-1. The *brc1* mutant has an early-flowering phenotype identical to the WT, whereas the transgenic *Arabidopsis* plants showed a late-flowering phenotype under LD (**Figures 3B, C**). To explore the mechanism by which *BjuBRC1*-1 influences flowering time, we tested whether BjuBRC1-1 could interact with BjuFT, similar to previous reports for *Arabidopsis* and hybrid aspen (Niwa et al., 2013; Maurya et al., 2020). Yeast two-hybrid, BiFC, and LCI assays showed that BjuBRC1-1 was capable of interacting with BjuFT *in vivo* but not in yeast (**Figure 4**). Based on these interactions between BjuBRC1 proteins and BjuFT, we speculated that BjuBRC1 proteins may delay flowering by interfering with the function of BjuFT. In rice, Hd3a is non-functional as a florigen without interacting with 14-3-3 proteins (Taoka et al., 2011). This is probably the reason that BjuBRC1 proteins were unable to interact with BjuFT in yeast. In addition, BjuBRC1 was capable of forming heterodimers, which thus probably enhanced its repressive influence on BjuFT (**Figure 5B**). Moreover, we found that BjuBRC1-1 protein (only full-length proteins) could bind to the promoter of *BjuFUL* directly to reduce its expression (**Figures 6**, **7A, B** **Supplementary Figure 11**). Moreover, *AtFUL* expression was reduced in transgenic *Arabidopsis* that ectopically expressed *BjuBRC1*-1 (**Figure 7C**).

Studies of floral organ development in model plants indicate that homologous genes controlling flower development are classifiable into five groups: classes A, B, C, D, and E (Weigel and Meyerowitz, 1994). *FUL* shows high similarity with the class A gene *APETALA 1* (*AP1*), has a conserved C-terminus, and is involved in the regulation of flower and fruit development in *Arabidopsis*. Most MADS-box genes are expressed only in flowers, whereas *FUL* is expressed at different specific stages of plant development (Coen and Meyerowitz, 1991; Borner et al., 2000; Drobyazina and Khavkin, 2006; Melzer et al., 2008). Thus, it is recognized that *FUL* plays an essential role in the control of flowering. Based on previous studies of *FUL*, we speculate that BjuBRC1-1 can directly repress *BjuFUL* expression to influence flowering or other growth-related processes in *B. juncea*.

Previous research has indicated that two *FT*-LIKE paralogs, *StSP3D* and *StSP6A*, control floral and tuberization transitions in potato (Navarro et al., 2011), and the orthologs of the CO and FT proteins have a conserved function in the control of tuberization in photoperiodic tuberization (Rodríguez-Falcón et al., 2006; González-Schain et al., 2012). A recent study found that *BRC1b* acts as a tuberization repressor in aerial axillary buds *via* interaction with SP6A, which prevents buds from competing in sink strength with stolons in potato (Nicolas et al., 2022). Based on the interaction between BjuBRC1s and BjuFT, we speculate that *BjuBRC1* may be involved in the regulation of tuberization in *B. juncea* (mustard), which requires further investigation.

**Figure 8:**
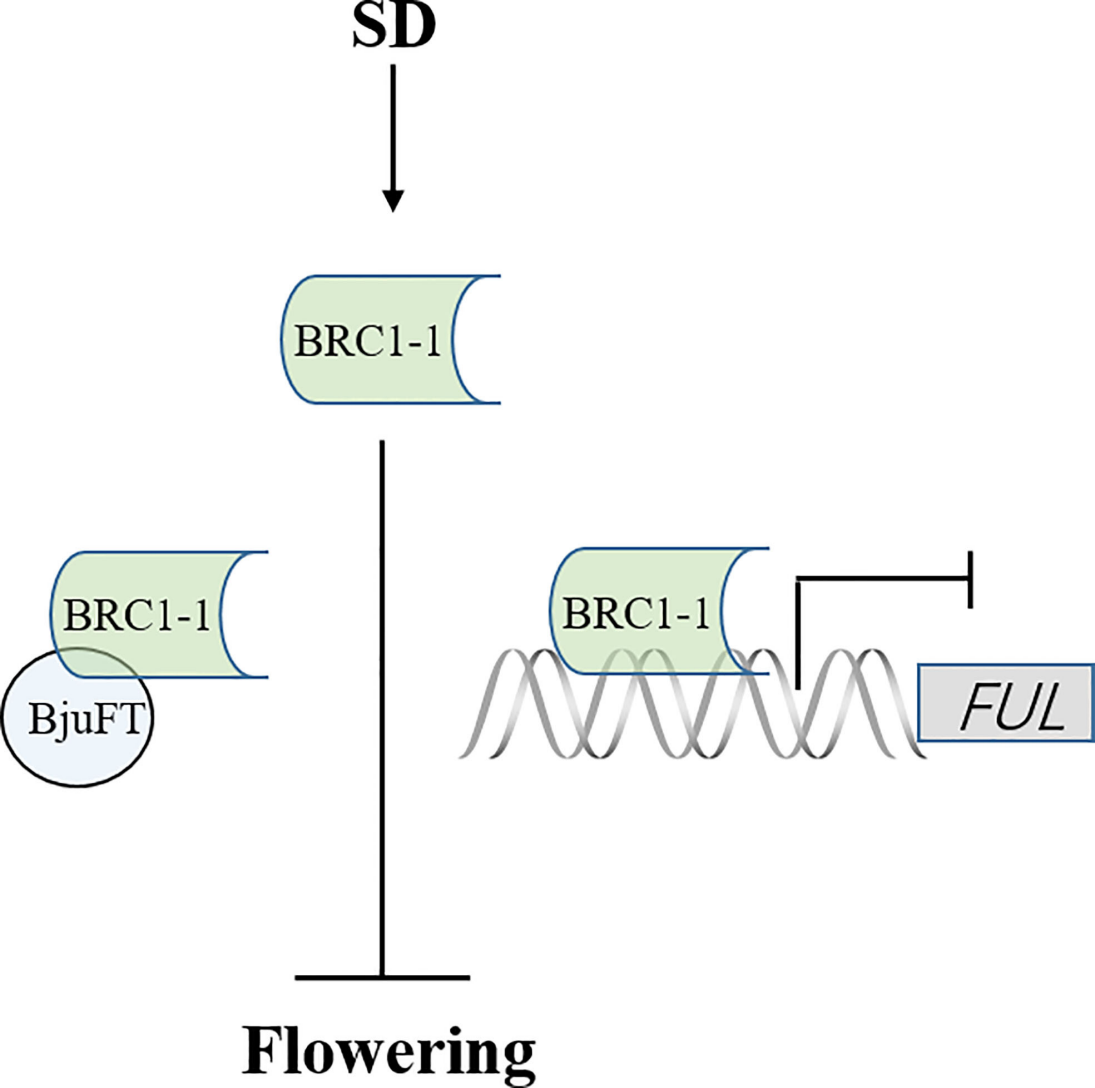
A proposed model of BjuBRC1-1 to regulate flowering. FT, FLOWERING LOCUS T; FUL, FRUITFULL; arrow, enhanced expression; horizontal line, inhibited expression.

The present study identified four homologous proteins of BjuBRC1 with distinct C-terminal and N-terminal regions in *B. juncea*. However, only BjuBRC1-1 could bind to the promoter of *BjuFUL*. Therefore, we speculate that the ability to bind specifically to the promoter of *BjuFUL* is conferred by the particular sequence in the N-terminal or C-terminal region. Interestingly, four homologous proteins could interact with BjuFT *in vivo*, and BjuBRC1-3 uniquely interacted in the cytomembrane and nucleus. Whether this mechanism is involved in the regulation of *B. juncea* development requires further study. Whether the five homologous *BjuBRC1* genes in *B. juncea* exhibit functional redundancy in the regulation of flowering or branching is currently unclear. In addition, it is possible that BjuBRC1 proteins interact with the promoters of other floral regulatory genes in addition to the *BjuFUL* promoter. In summary, a proposed model of BjuBRC1-1 to regulate flowering is established (**Figure 8**). And the present results provide evidence that BjuBRC1 genes may be involved in the regulation of flowering and provide a theoretical basis for breeding mustard cultivars with a specific desired flowering and maturity time.

## Data availability statement

The raw data supporting the conclusions of this article will be made available by the authors, without undue reservation.

## Author contributions

QT and JF designed the study. QT, JF, QD, and HL performed the experiments and analyzed the data. JF wrote the paper. QT, QD, HL, DW, and ZW revised the manuscript. QT is the corresponding author. All authors contributed to the article and approved the submitted version.

## Funding

This work was funded by grants from the Natural Science Foundation of Chongqing (cstc2019jcyj-zdxmX0022) and the Technology Innovation and Application Development Project of CQ CSTC, China (cstc2021jscx-gksbX0021).

## Conflict of interest

The authors declare that the research was conducted in the absence of any commercial or financial relationships that could be construed as a potential conflict of interest.

## Publisher’s note

All claims expressed in this article are solely those of the authors and do not necessarily represent those of their affiliated organizations, or those of the publisher, the editors and the reviewers. Any product that may be evaluated in this article, or claim that may be made by its manufacturer, is not guaranteed or endorsed by the publisher.
