# Supplementary material for: Brassica juncea BRC1-1 induced by SD negatively regulates flowering by directly interacting with BjuFT and BjuFUL promoter
Source: Front Plant Sci. 2022 Sep 30;13:986811. doi: 10.3389/fpls.2022.986811 (PMC9561848; doi:10.3389/fpls.2022.986811)
Supplement: Supplementary file 1 [file DataSheet_1.docx]

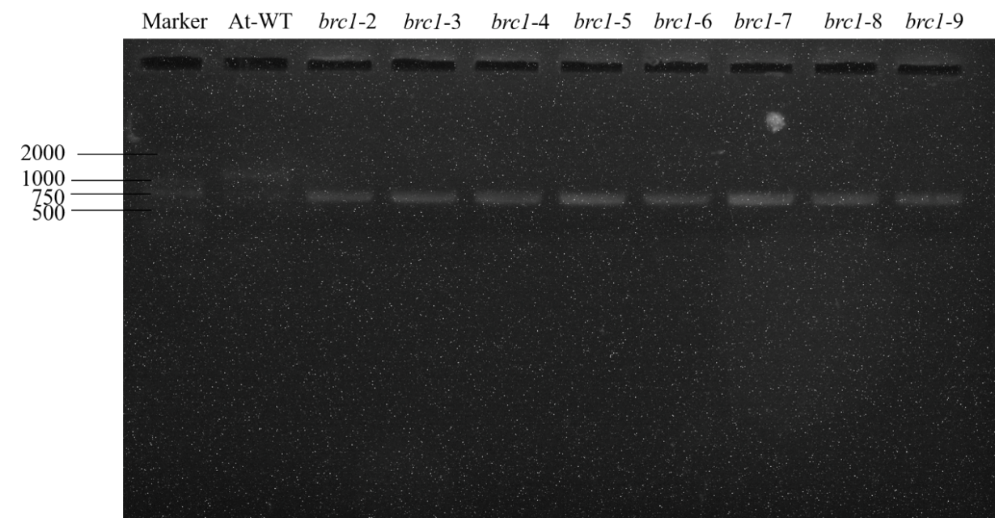


**Supplementary Figure 1 |** PCR amplify to detect positive *brc1 Arabidopsis* (T-DNA insert lines, three primers method).


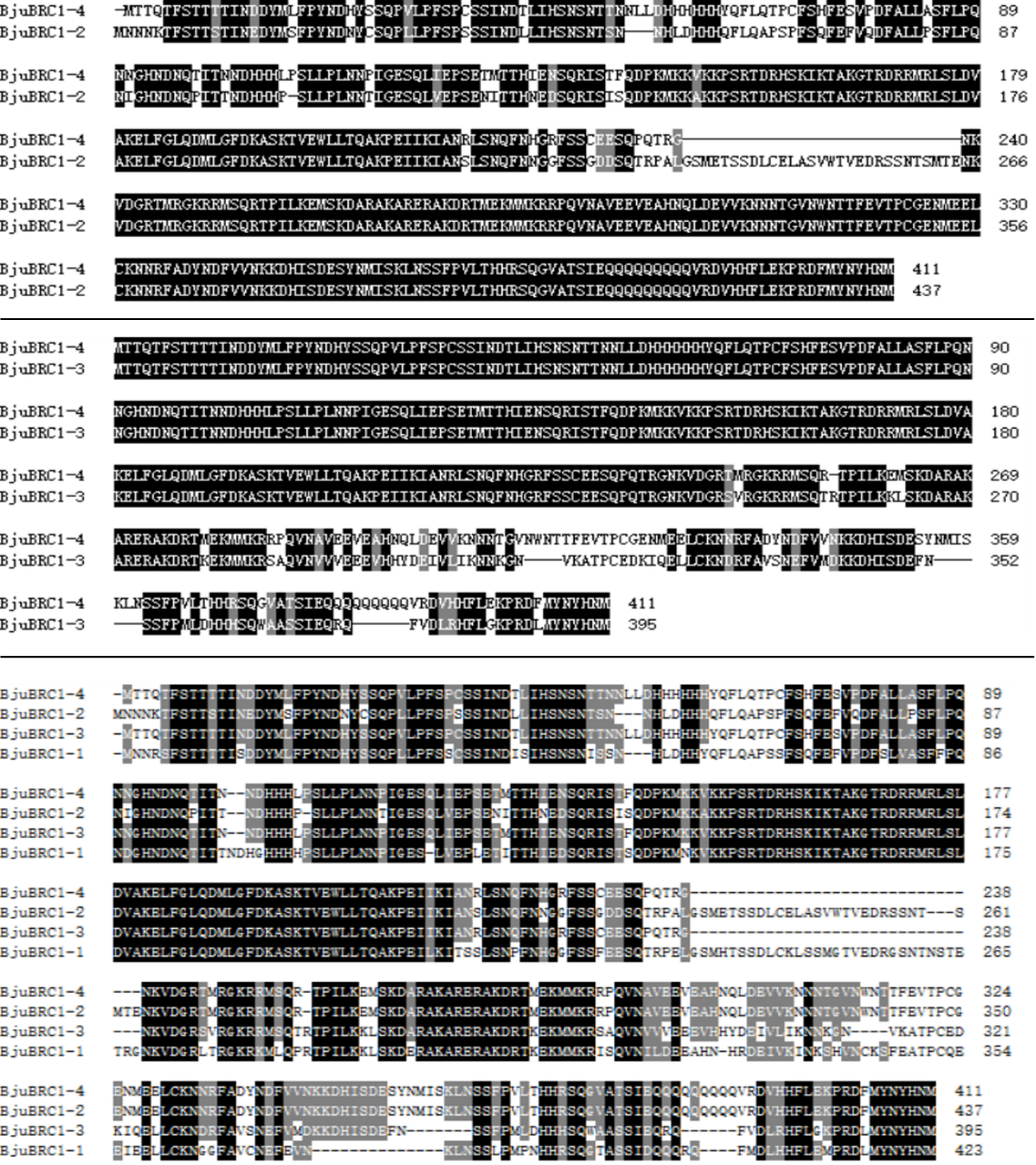


**Supplementary Figure 2 |** Proteins sequence alignment of BjuBRC1s.


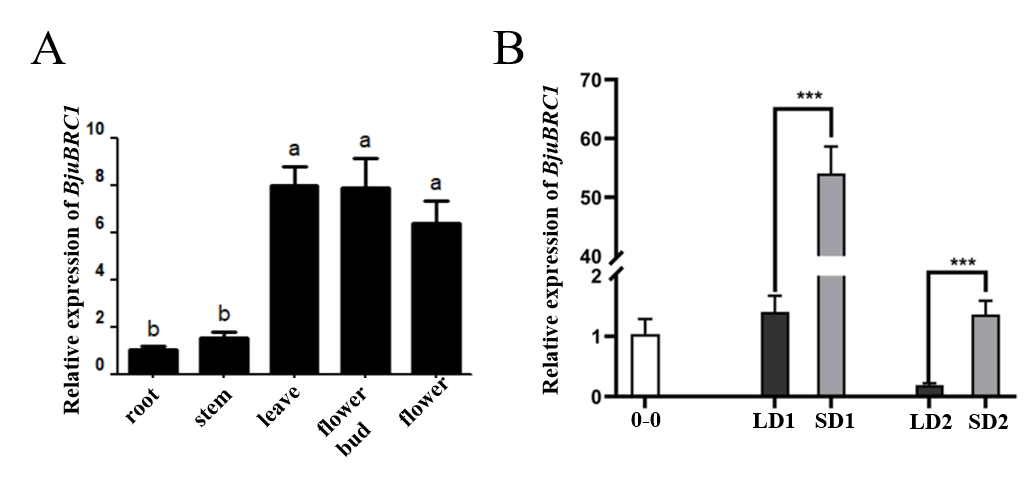


**Supplementary Figure 3 |** The expression analysis of *BjuBRC1*-1 genes in *B. juncea*. **(A)** Relative expression levels of *BjuBRC1*-1 in different tissue. Different letters indicate significant differences between means as determined by an ANOVA followed by Duncan’s multiple range test (P < 0.05). **(B)** Relative expression levels of *BjuBRC1*-1 in different developmental stages (0, 1 and 2, two adjacent stages are separated by 14 days) under long-day (LD1 and LD2) and short-day (SD1 and SD2), respectively. “0-0” indicates plant materials growth 10 days after germination under neutral-days condition. “***” indicates significant difference, p < 0.001, by Student’ s t-test. Error bars represent SE.


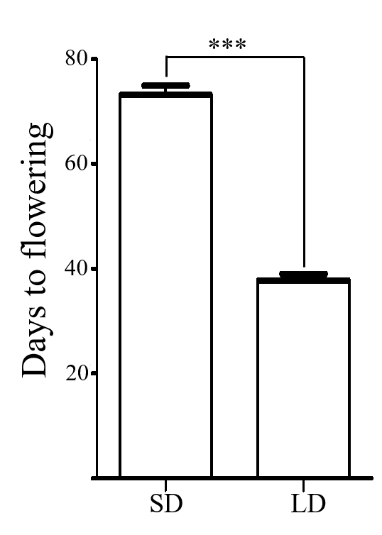


**Supplementary Figure 4 |** Days to flowering of *B. juncea* under long-day (LD) or short-day (SD) conditions. Error bars represent SE. “***” indicates significant difference, p < 0.001, by Student’ s t-test.


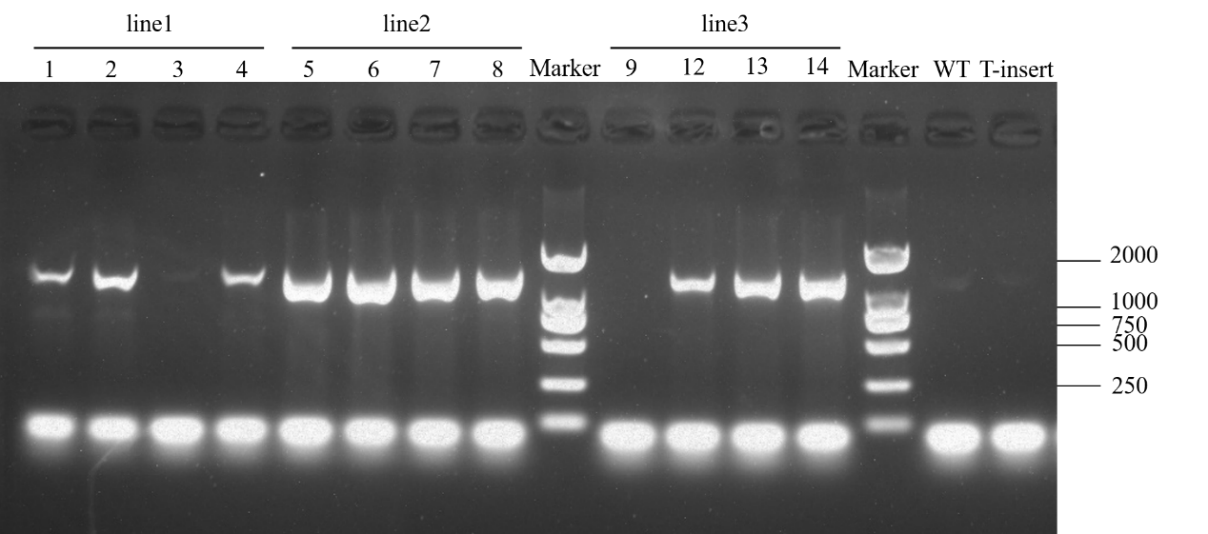


**Supplementary Figure 5 |** Identification of T_3_ transgenic *Arabidopsis* plants, and primers used for the PCR amplification are gene clone primers with *Xba* Ⅰ (F) and *Kpn* Ⅰ (R).


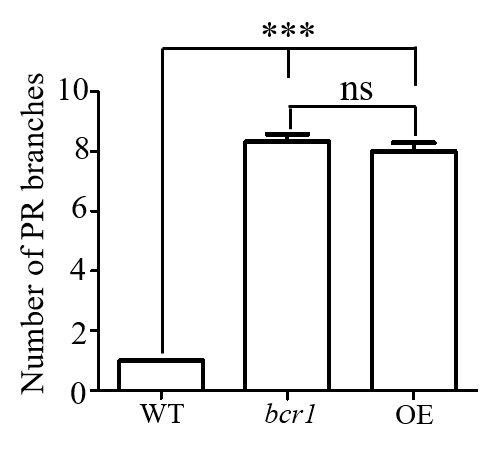


**Supplementary Figure 6 |** Branches of the WT, *brc1* and the transgenic *Arabidopsis* (OE) while one week after flowering. Error bars represent SE. “ns” indicates no significance, “***” indicates significant difference, p < 0.001, by Student’ s t-test.


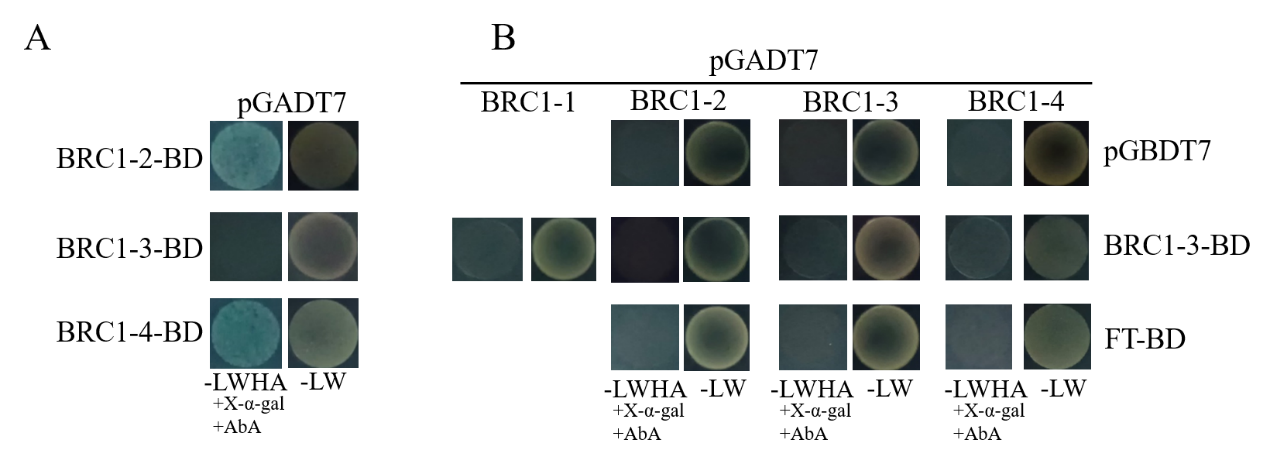


**Supplementary Figure 7 |** Yeast two-hybrid assays to detect interactions. **(A)** Self-activation detections of three BjuBRC1 homologous proteins. **(B)** Yeast two-hybrid assay. Clones containing each combination of bait and prey vectors were grown on nonselective medium (- X-α-gal/ - AbA) or on selective medium (+ X-α-gal/ + AbA), pGBDT7 empty vectors were used as a negative control (AD, activation domain; BD, binding domain respectively).


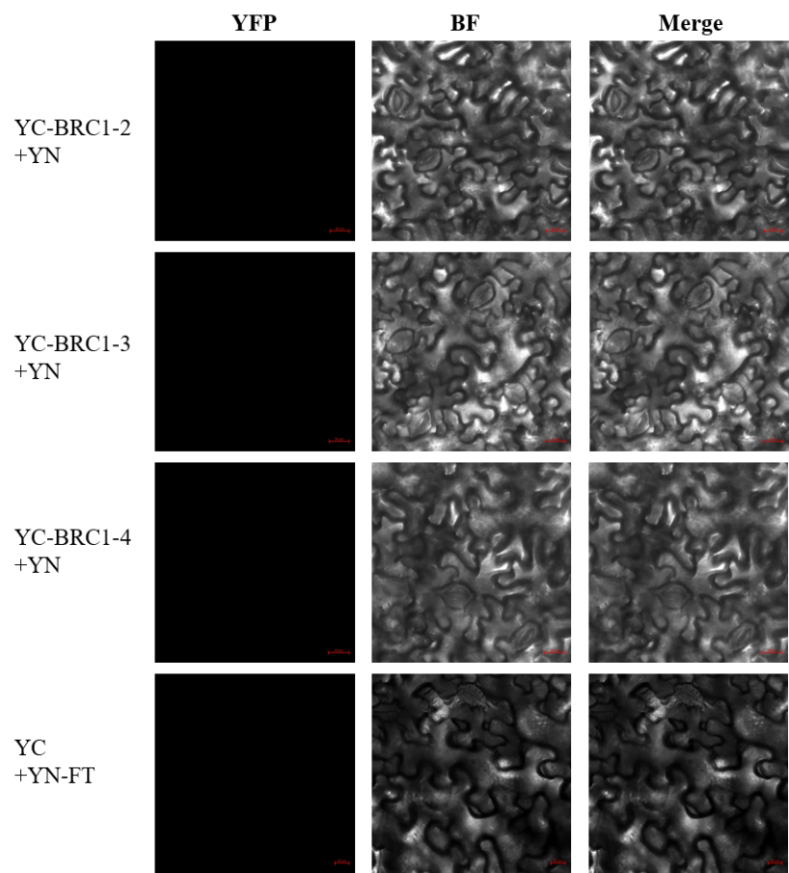


**Supplementary Figure 8 |** BiFC assay between three BjuBRC1 proteins and BjuFT (negative control)**.**


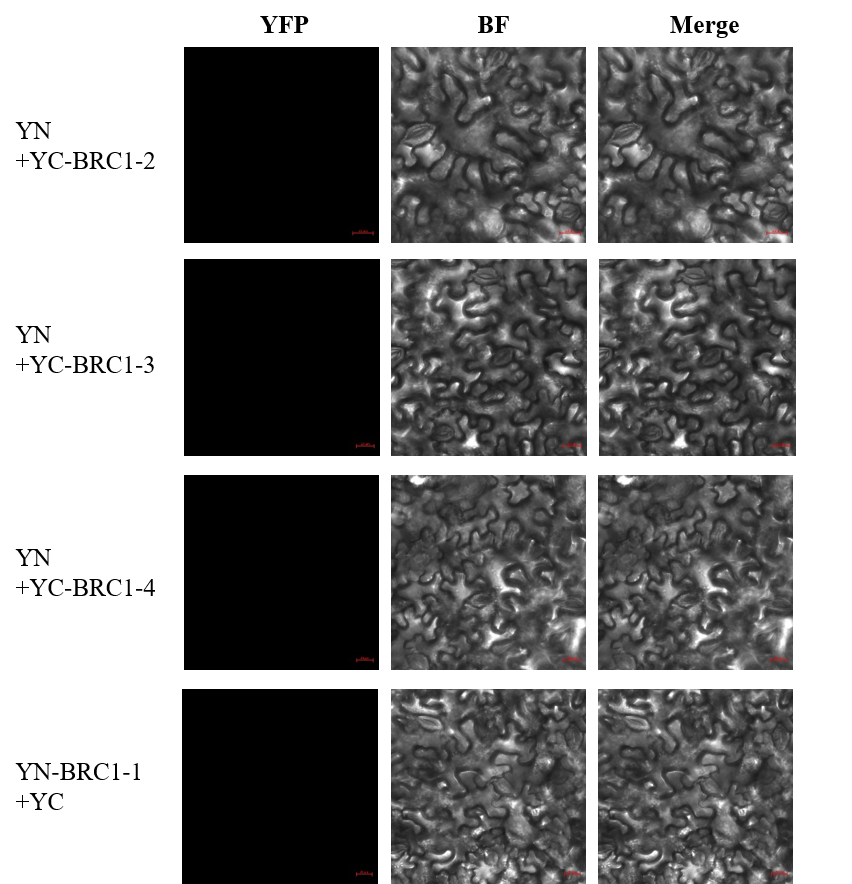


**Supplementary Figure 9 |** BiFC assay between BjuBRC1-1 and BjuBRC1-2, BjuBRC1-3 or BjuBRC1-4 (negative control).


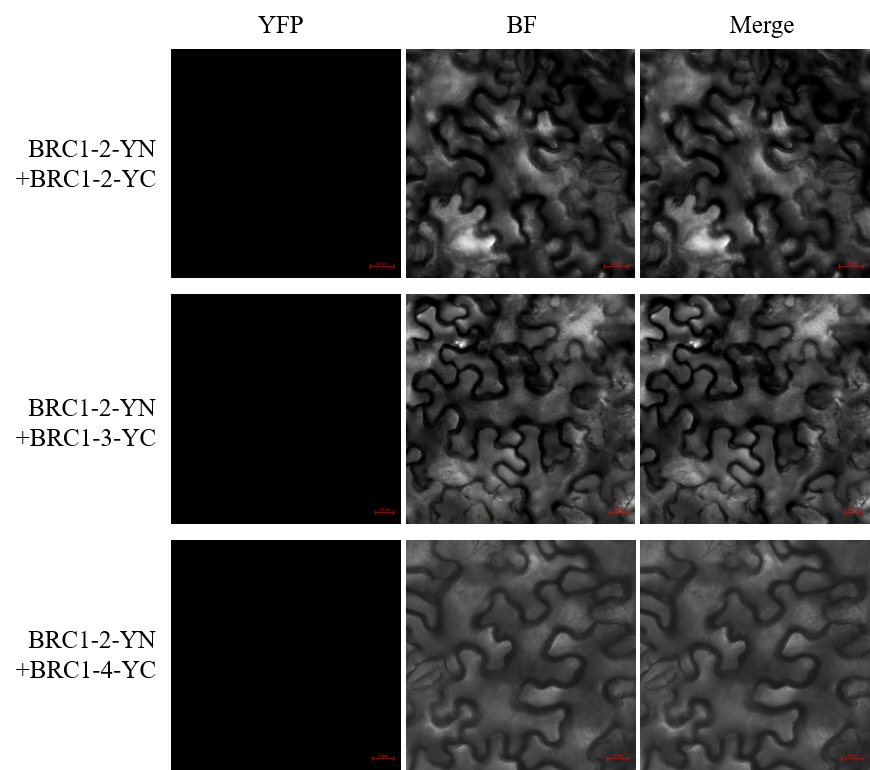


**Supplementary Figure 10 |** BiFC assay to detect whether BjuBRC1-2 could form heterodimers with BjuBRC1-3 or BjuBRC1-4, and homodimer.


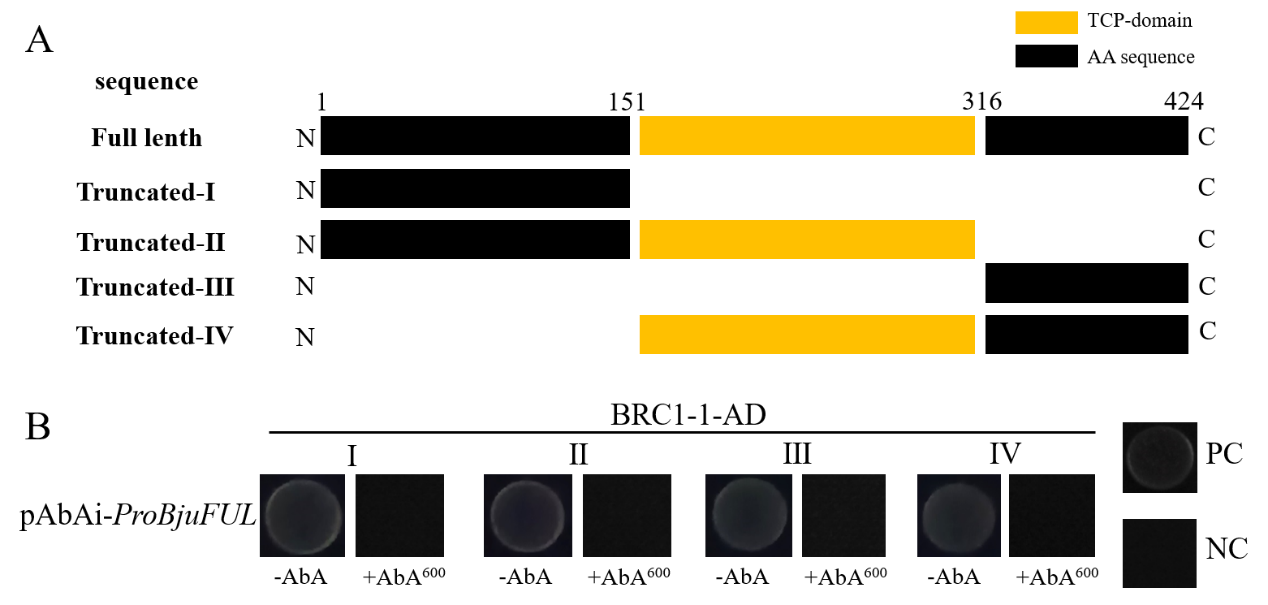


**Supplementary Figure 11 |** Detections of interactions between four truncated proteins of BjuBRC1-1 and *ProBjuFUL* via yeast one-hybrid assay. **(A)** Schematic diagram of four truncated proteins. **(B)** Yeast one-hybrid assay to test whether the four truncated BjuBRC1-1 proteins could directly bind to *BjuFUL* promoters, respectively. pGADT7-BjuBRC1-1-Ⅰ (Ⅱ, Ⅲ and Ⅳ, respectively) was transformed into yeast Y1H strains in combination with pAbAi-*ProBjuFUL*. The transformed strains all were grown on SD/-Leu selective media containing 600μg/L aureobasidin A (AbA). PC, positive control; NC, negative control.

| **Name**  **BRC1-F1**  **BRC1-F2**  **BRC1-R**  **BjuFT-F**  **BjuFT-R**  **At-BRC1-LP**  **At-BRC1-RP**  **LBb1.3**  **BRC1-Ⅰ-R**  **BRC1-Ⅱ-R**  **BRC1-Ⅲ-F**  **BRC1-Ⅳ-F**  **BRC1-qPCR-F**  **BRC1-qPCR-R**  **BRC1-1300-Ⅰ(Ⅱ)-F**  **BRC1-1300-Ⅰ-R**  **BRC1-1300-Ⅱ-R**  **BjuACTIN2-qPCR-F**  **BjuACTIN2-qPCR-R**  **ProBjuFT-F**  **ProBjuFT-R**  **ProBjuFUL-F**  **ProBjuFUL-R**  **ProBjuSOC1-F**  **ProBjuSOC1-R**  **AtFUL-qPCR-F**  **AtFUL-qPCR-R**  **AtTUB2-qPCR-F**  **AtTUB2-qPCR-R** | **Sequences (5′→3′)**  **ATGAACGACAACAAGACTTTC**  **ATGAACAACAGGTCTTTCAGT**  **TCACATATTATGGTAGTTGTACATGA**  **AATAGAGATCCTCTTGTGGT**  **CTAGCTTCTTCGWCCTCCG**  **TGTAGAACAACCCACTGAGCC**  **ATCGATGGTGGTGCATTAGTG**  **ATTTTGCCGATTTCGGAAC**  **CGCGGATCCTTTCTTGACTTTATTC**  **CGCGGATCCCCTCTTCATCATCTTCTCC**  **CGCCATATGATATCACAAGTAAAT**  **CGCCATATGCCAAGCAGAACGGA**  **GGACAGCCAGCTCAATAGATCA**  **TGTACATGAGGTCTCTTGGCATC**  **GCTCTAGAATGAACAACAGGTCTTTCAGTAC**  **GGGGTACCTTTCTTGACTTTATTC**  **GGGGTACCCCTCTTCATCATCTTCTCC**  **GCTGACCGTATGAGCAAAGA**  **GTTGGAAAGTGCTGAGGGAT**  **TGTCTATATATTGATGCATTGTC**  **ATTTGATCTAAAACAAACAGGTGG**  **GTTGACCCAACTAAAGTTGAAA**  **TTCAAAATTGTAGAATAACTTTCT**  **GAAAGGAGAGTGTGTATGTGTTGTC**  **AAGGCTTTCTTCAGCAAACCATTCC**  **CGATGCTGAGGTTGCTCTCA**  **ACTTTGTGAAACGTCTCGGC**  **ATCCGTGAAGAGTACCCAGAT**  **AAGAACCATGCACTCATCAGC** |
| --- | --- |

**Supplementary Table 1 |** Primers used in the paper.
